# Comparative review of human and canine osteosarcoma: morphology, epidemiology, prognosis, treatment and genetics

**DOI:** 10.1186/s13028-017-0341-9

**Published:** 2017-10-24

**Authors:** Siobhan Simpson, Mark David Dunning, Simone de Brot, Llorenç Grau-Roma, Nigel Patrick Mongan, Catrin Sian Rutland

**Affiliations:** 1 Faculty of Medicine and Health Sciences, School of Veterinary Medicine and Science, The University of Nottingham, Sutton Bonington Campus, Loughborough, LE12 5RD UK; 2 Department of Pharmacology, Weill Cornell Medicine, 1300 York Avenue, New York, NY 10065 USA

**Keywords:** Bone cancer, Canine, Genetics, Human, Molecular diagnostics, Osteosarcoma, Treatment

## Abstract

Osteosarcoma (OSA) is a rare cancer in people. However OSA incidence rates in dogs are 27 times higher than in people. Prognosis in both species is relatively poor, with 5 year OSA survival rates in people not having improved in decades. For dogs, 1 year survival rates are only around ~ 45%. Improved and novel treatment regimens are urgently required to improve survival in both humans and dogs with OSA. Utilising information from genetic studies could assist in this in both species, with the higher incidence rates in dogs contributing to the dog population being a good model of human disease. This review compares the clinical characteristics, gross morphology and histopathology, aetiology, epidemiology, and genetics of canine and human OSA. Finally, the current position of canine OSA genetic research is discussed and areas for additional work within the canine population are identified.

## Background

Cancer is a leading cause of non-communicable morbidity and mortality throughout the world, second only to cardiovascular disease in the number of deaths of adults between the ages of 30 and 70 years old [1, 2]. For dogs, the most frequent causes of mortality in order are (i) cancer, (ii) trauma, (iii) locomotor (skeletal, muscles and joints), (iv) cardiac disease, and (v) neurological disease [3]. The development of new and superior cancer treatments has long been a priority in medicine, more recently there has been a drive to develop new approaches to treat and manage cancer in companion animals. Indeed, cancer in dogs is increasingly a concern for pet owners, in particular as some types of cancer occur at much higher rates in certain dogs breeds [4–7]. These cases of cancer in dogs themselves require treatment, in addition these spontaneous cancers in dogs represent effective models for the human disease equivalent [8]. Although developing new treatments in companion animals with naturally occurring disease is less contentious than inducing disease in experimental animals, ethical concerns with regards to the treatment of individuals enrolled on studies and gaining informed consent from owners remain [9].

Cancer is more common in adults than children, adolescents, and young adults (< 30 years old) [10, 11]. Despite this, bone cancer is most common in the young (< 20 years old) and elderly (over 60 years old) than individuals of intermediary age (20–60 years old) [10–12]. For many cancer types 5 year survival rates are improving, reflecting the development of earlier diagnostic techniques and improved treatments [1, 13]. In contrast to many other cancers OSA, the most common bone cancer, has not shown comparable improvements in mortality rates [13–15]. There is thus an urgent need to develop improved treatments for OSA.

In both human and canine patients the predominant bone cancer diagnosis is OSA [15, 16]. OSA is still considered rare in humans. For example, there were 217,440 estimated breast cancer cases in the NIH–NCI surveillance, epidemiology, evidence and end results (SEER) database in 2004 alone, compared to 3482 cases of OSA in the same database between 1973 and 2004 [15, 17]. Although OSA is also not very common in dogs, it is much more common than in people. Norwegian owner questionnaire data from four breeds (Labrador retriever, Newfoundland, Leonberger and Irish wolfhound) showed OSA incidences of between 0.2 and 8.9% depending on the breed [18]. The percentage of dogs positively identified with OSA in two Polish clinics was estimated to be between 2.7 and 10.7%, again depending on breed [19]. Between 1995 and 2002 of 394,061 insured Swedish dogs, 764 (0.19%) developed a bone cancer [16]. This is an incidence rate of 27.2 dogs per 100,000 included in the dataset each year. While the canine data only includes insured dogs, it is a large dataset so is likely to be representative of the dog population of the country as whole. In the comparable Swedish population, 234 men and 164 women had bone cancer between 1998 and 2002, which is roughly 0.89 cases of bone cancer per 100,000 people each year [11]. The human data records all bone cancer cases in the country and the average population size between 1998 and 2002 was calculated from government census data. In addition all other countries in the “Cancer Incidence in Five Continents” registry report human incidence rates similar to Sweden [11, 12]. Thus, the incidence of canine bone cancer is 27 times higher than in humans. The higher incidence rate of canine OSA makes the pet dog population a good model for human disease. While there is potential for any dog to develop OSA, a subset of larger dog breeds are at increased risk of developing OSA [16].

The purpose of this review is to give an insight into the morphology, epidemiology, prognosis, treatment and genetics of osteosarcoma and to compare these aspects, in light of the published literature, between humans and dogs. Such knowledge is required to translate advances made in the clinical management of OSA in people to dogs; and to advance our understanding of where disease processes are similar across species.

## Search strategy

This review is based on a search in PubMed (http://www.ncbi.nlm.nih.gov/pubmed) using the terms “canine, dog, human, osteosarcoma, cancer, tumour, oncology”. Each title and abstract of the obtained hits were evaluated and articles referring to genetics, epidemiology, treatment, histology and prognosis were assessed in detail (full text). Searches were carried out from 06/2016 to 02/2017 an additionally 08/2017. All titles, abstracts and full texts were read and obtained by all authors. Searches included web of science and PubMed. All papers published prior to, and including 08/2017 were included. Our personal photograph archives were used to illustrate OSA (with appropriate ethical permissions) and our experience within the field was used to evaluate the literature.

## Gross and histological similarities and differences between canine and human OSA

**Fig. 1:**
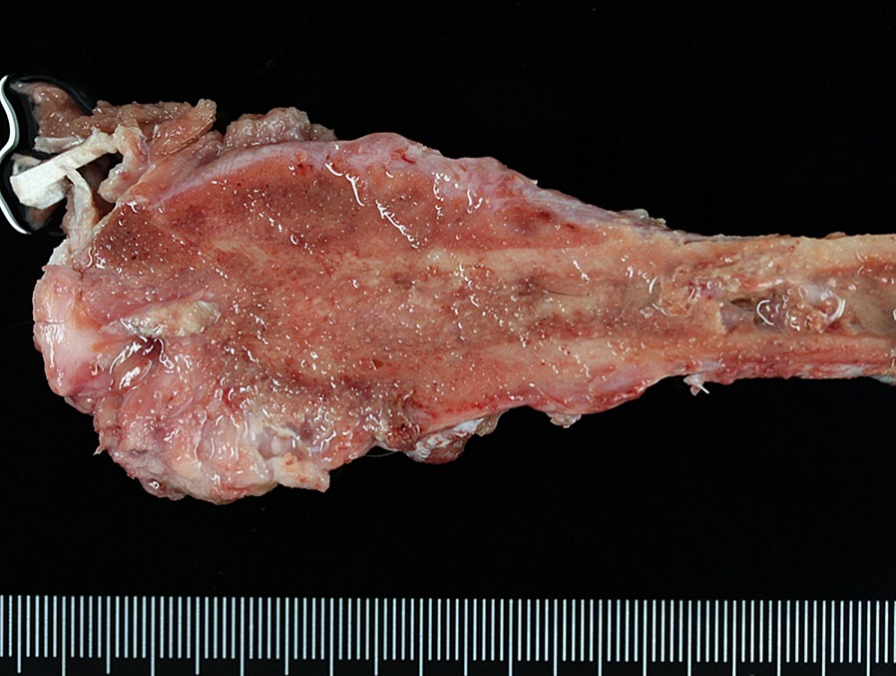
Femoral osteosarcoma. Greyhound, 7 years and 7 months old. Dense sclerotic neoplastic proliferation with hard consistency at the level of the proximal metaphysis. The tumour infiltrates the medullary cavity and transgresses the cortical bone

**Fig. 2:**
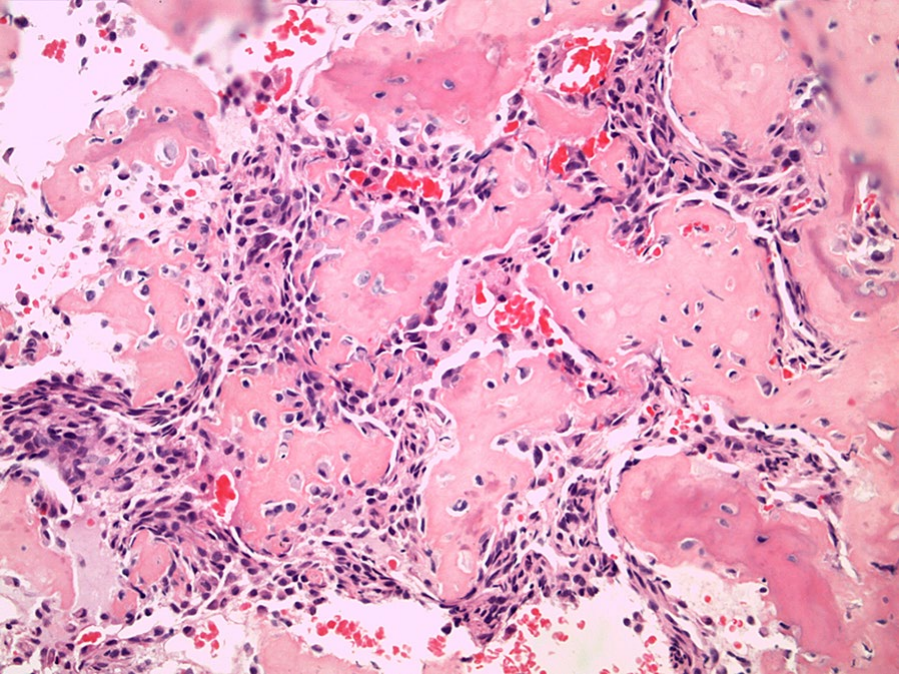
Microphotograph of a productive osteoblastic osteosarcoma. Abundant accumulation of extracellular lakes of osteoid between malignant osteoblasts. Haematoxylin and eosin stain. Obj. 20x

A sarcoma is a malignant tumour originating in tissues derived from the mesoderm; affecting bone, cartilage and other connective tissues [20]. OSA is a sarcoma which produces bone or osteoid [21]. In both people and dogs, the gross appearance of the OSA is markedly variable, some being predominantly lytic (soft, fleshy and with areas of haemorrhage and necrosis), productive (hard consistency and variably grey in colour), or being a mix of both. It frequently transgresses the cortex at the same time that it grows within the medulla, rarely penetrating the joint (Fig. 1) [22]. World Health Organisation (WHO) defines OSA as a primary malignant bone tumour in which the neoplastic cells produce osteoid [22]. In both people and dogs, OSA is characterized by a highly pleomorphic and heterogeneous microscopic appearance, and it is divided into several histologic subtypes similar in both species (Table 1) [22, 23]. Commonly, these subtypes are typically mixed in the same tumour, which questions the significance of histologic tumour classification for prognostic purposes. Indeed, histologic subtype could not be demonstrated to influence biological tumour behavior in dogs [24] and is controversially discussed in human literature. The most common subtype is the osteoblastic form in both people and dogs (Fig. 2) [21, 25, 26]. Furthermore, OSAs are classified on tumour grade, which is based on microscopic features such as cellular pleomorphism, mitotic index, tumour matrix and degree of necrosis [25–27]. The vast majority of canine and human OSAs are high grade tumours [21, 26, 28]. Regarding the prognostic effect of OSA grading, the veterinary literature remains divided [26, 29, 30], whereas it is considered important and relevant for prognosis in human medicine [27].

**Table 1:** Histologic subtypes of human and canine osteosarcoma

Species	Canine^a^	Human^b^
Subtypes	A. Central	Osteosarcoma
	Osteosarcoma	Low-grade central osteosarcoma
	a. Poorly differentiated	Conventional
	b. Osteoblastic	Chondroblastic
	Nonproductive	Fibroblastic
	Productive	Osteoblastic
	c. Chondroblastic	Secondary
	d. Fibroblastic	Telangiectatic
	e. Telangiectatic	Small cell
	f. Giant cell type	Parosteal
	B. Peripheral	Periosteal
	1. Periosteal osteosarcoma	High grade surface
	2. Parosteal osteosarcoma	

^a^Based on World Health Organization (WHO) International Histological Classification of Tumors of Domestic Animals [23]

^b^Based on WHO Classification of Tumours of Bone [22]

The vast majority of OSAs in both dogs and humans arise from within bones, particularly in the metaphyseal regions of long bones [22, 23] but can also originate on the surface of the bone or be extra-osseous [21]. In both humans and large dogs, OSA has a clear preference for the appendicular skeleton (up to 80% in dogs and 90% in humans) over the axial skeleton, with different bone percentages of involvement associated to the main weight bearing regions in each species. In people, ~ 50% of cases occur in the region of the knee (distal femur > proximal tibia followed by proximal humerus in approximately 25% of cases), in dogs the forelimbs are affected twice as often as the hind limbs (mainly distal radius and proximal humerus followed by distal and proximal femur and distal tibia) [31].

## Treatment of osteosarcoma in humans and dogs

In both humans and dogs, treatment for OSA involves surgery to remove primary tumours and on occasion distant metastasis, combined with neoadjuvant and/or adjuvant chemotherapy [32, 33]. Surgery involves either amputation of the limb or limb salvage/sparing procedures [34, 35]. Limb salvage is most frequently performed on patients with lower grade tumours and has been associated with similar outcomes to conventional approaches using amputation [35]. The proposed advantage of limb salvage surgery over amputation is that these patients have a better return-to-function and consequently quality-of-life than those undergoing amputation [36]. A recent meta-analysis supported this benefit in addition to a lower incidence of metastasis with limb salvage [37]. Similar advantages have yet to be reported in dogs undergoing limb salvage, perhaps suggesting poor cross-species efficacy. Canine reports suggest a similar metastatic frequency and survival time with limb salvage compared with the more conventional approach of amputation and adjuvant chemotherapy [38]. The failure to identify a significant difference in the outcomes in dogs compared with humans may be related to fewer numbers of dogs undergoing limb salvage procedures and therefore a more limited experience with the procedure. It is also possible that differences in case selection between humans and dogs impacts on outcome, in dogs clearly this may be the result of both patient factors and owner factors. Until a greater number of cases appear in the canine literature, the reason for the differences in outcomes remains unclear. Of note however from the canine literature on limb salvage, is that post-operative infections are associated with increased survival times [39–42]. The mechanism responsible for this prolonged survival and disease-free interval has yet to be elucidated, but is hypothesised to be due to up-regulation of antitumor immunity [39]. This is phenomenon is not unique to the dog and has also been reported in humans, with a similarly unclear aetiology [43, 44].

In human OSA, surgery with adjuvant chemotherapy remains the treatment of choice [45, 46]. The standard adjuvant chemotherapeutic approach uses a combination of methotrexate, doxorubicin and cisplatin using either a high, moderate, or standard dose protocols [47–50]. This approach has remained relatively unaltered for many years. The inclusion of additional chemotherapeutics including ifosfamide has met with mixed results and meta-analyses have provided conflicting evidence as to whether this provides a clear benefit in all cases [51, 52]. Whilst a large number of protocol modifications and experimental therapies have been reported, these at present do not offer credible therapeutic alternatives to improve outcome in affected individuals [49]. However there is evidence suggesting six doses of carboplatin may be superior to alternating doxorubicin and carboplatin regimens [53]. In canine OSA adjuvant chemotherapy with either doxorubicin or the platinum-based compounds including cisplatin and carboplatin have been associated with increased survival times over amputation alone [54–57]. The relative merits of the individual chemotherapy drugs have been evaluated. Reports indicate no significant difference in disease free interval or survival times in those dogs receiving either post-operative doxorubicin or carboplatin or combinations thereof for OSA [32]. However, those dogs receiving carboplatin experienced fewer side-effects improving the quality of life during therapy, an important consideration for most owners. Combination protocols with doxorubicin and carboplatin have also been evaluated for their potential benefit in improving disease free interval and survival times [58]. This combination protocol has been associated with a greater degree of chemotherapy induced toxicity [59]. A recent prospective study comparing carboplatin with an alternating carboplatin-doxorubicin protocol showed no benefit of the combination in improving survival over carboplatin alone [53]. Other (retrospective) publications using this combination have failed to demonstrate a clear benefit over single-agent carboplatin [58, 59]. Limited publications exist evaluating the benefits of additional chemotherapeutic agents for canine OSA e.g. gemcitabine; none provide a clear improvement in outcome above the use of carboplatin [60].

The use of bisphosphonate therapy as an adjunct to standard approaches for treating OSA has received reasonable coverage in the literature. A wide variety of experimental data exists on their efficacy in vitro and in models of OSA [61–65]. There has however been equivocal clinical benefit from their use in both in vivo models and in naturally occurring disease [66–68]. Whilst their value in management of the primary disease is unclear, perhaps more compelling evidence exists for their role in managing metastatic disease [63, 69–71]. This is a very important feature in managing OSA, given the importance of metastatic disease in overall mortality.

More recent reports on the use of immunotherapy based on an attenuated recombinant *Listeria monocyotogenes* expressing a chimeric HERS/neu fusion protein have shown some encouraging results [72]. Dogs having undergone surgery (with amputation or limb salvage) with post-operative chemotherapy with four doses of carboplatin were enrolled in this study. In these dogs, 3 weeks after completion of their chemotherapy protocol they received the immunotherapy agent. There was an overall increase in survival time and reduction in the incidence of metastasis compared with historic controls. Newer therapeutic treatments including immunotherapies are also being investigated and developed in people given the failure to improve the outcome of human patients significantly in the past 30 years [73–75].

Given the failure to significantly improve the treatment approach and therefore outcome of OSA in humans over the past 3 decades [76, 77] and in dogs over the same period [78–80], novel approaches are clearly required. One particular area of medicine that may provide improved treatment approaches for OSA focuses on the field of pharmacogenomics. Here specific pharmacogenetic biomarkers and targets can provide a personalised approach to therapy for OSA [81, 82]. This approach whilst desirable remains in its infancy but clearly provides exciting potential for future management of OSA in both humans and dogs.

## Prognostic factors for osteosarcoma in humans and dogs

Key factors affecting treatment outcome are diagnostic and prognostic tools. Typical OSA disease progression consists of a primary tumour, usually originating within the medullary cavity, which grows, proliferates, and invades, and left unchecked frequently metastasises to the lungs [83]. Prognosis for individuals with metastatic tumours is much poorer than for individuals with only primary tumours. The 5-year-event-free-survival for humans with metastatic tumours at diagnosis reported to be 27.4%, while individuals with no metastases at diagnosis the 5-year-event-free-survival is ~ 70% [15, 83]. The prognosis for canine OSA is poor with 1 year survival rates following diagnosis and treatment typically less than 45% [84–86]. Of those dogs surviving beyond 1 year, ~ 54% will develop metastatic disease with a median survival time of 243 days [40]. In dogs with evidence for metastatic disease at the time of diagnosis, the median survival time is 76 days [23]. There is gathering evidence in some cases (although limited by study numbers) that in addition to the understanding that certain breeds are predisposed to developing OSA, there are also specific genetic differences within the tumours encountered in certain breeds which may have prognostic significance [87, 88].

A number of factors have been suggested to have prognostic significance in canine OSA. Bodyweight and tumour location have been suggested to be of particular importance and are readily available parameters for all patients. Dogs with lower body weight have a longer survival time [39, 89, 90] and dogs with proximal humeral tumours experience a shorter survival time [91]. Serum biomarkers have also been evaluated, such markers are clearly desirable particularly if routinely measured as this helps clinicians to begin discussions around prognosis with clients without recourse to advanced diagnostics. Increased serum alkaline phosphatase activity in dogs has been associated with a shorter disease free interval and survival time [92]. Histological grading is relevant for prognosis in human medicine [27], but remains controversial in dogs [26, 29, 30]. A recent meta-analysis in dogs has suggested that ALP and a proximal humeral location are significant negative prognostic factors. The importance of other factors was difficult to determine in this meta-analysis due to limited numbers of dogs and studies in which these had been reviewed [91]. In humans, the extent of any histological response to pre-operative chemotherapy has significant predictive value for both local and distant disease control [48, 77]. A similar effect has not been reported in dogs, this may be due to pre-operative chemotherapy not being part of routine treatment for canine OSA. In both humans and dogs molecular and genetic factors have also been identified as having potential prognostic significance. Many of these have been identified in OSA cell lines and therefore their clinical significance needs clarifying, however a number of cell lines have shown similarities between human and canine OSA, these therefore represent a very valuable evaluation tool [93]. Some of the factors observed in cell lines include HER-2 and PTEN, which have also been identified in some tissue samples [31]. The expression of matrix metalloproteases have also been demonstrated in some tissue samples (MMPs 2 and 9), however the clinical and prognostic significance of these is unclear. In humans, the literature often has conflicting data on the significance of particular markers. For example, the human epidermal growth factor 2 oncogene (HER-2) has been suggested in some reports to have prognostic significance, though further study is required to definitively confirm this [94–96]. Epidermal growth factor has been evaluated in dogs, whilst expressed in primary and metastatic tumour cells, it has not been shown to be an effective prognostic marker clinically [97]. Vascular endothelial growth factor A (VEGFA) has also been implicated in prognosis and over-expression has been associated with shorter survival times in humans [98]. However, at present the actual value of VEGFA in prognostication remains to be clarified [98, 99]. In humans p53 has been shown to be an effective prognostic marker and upregulated p53 is associated with a shorter survival time [100]. This has also been associated with prognosis canine OSA [101]. Strong expression of CXCL-12 is also associated with a more favourable outcome in humans with OSA [96]. The significance of CXCL-12 expression has yet to be investigated in dogs. MicroRNA transcripts have also been investigated for their prognostic significance. It would seem that down-regulation of 14q32 miRNA expression is a conserved mechanism in both human and canine OSA samples and influences tumour behaviour [102]. One particularly promising molecule however is ezrin, a membrane cytoskeleton linking molecule. This has been associated with a shorted survival time in both humans and dogs with OSA [103–106]. In addition to overall prognosis, a number of factors have been implicated in the development of metastatic disease. Given that local disease control is not the major life-limiting aspect to both canine and human OSA, identifying factors affecting metastasis are crucial. A number of markers have been identified in humans including Hes4 [107] and in canine OSA the proto-oncogene c-Met has been implicated in lymphatic spread [108].

## Epidemiology and genetics of osteosarcoma in humans

Some cases of human OSA are associated with heritable cancer syndromes, and the genetic bases of these have been established [109–111]. Most instances of OSA, however are not associated with heritable cancer syndromes and there have only been two heritable genetic loci identified, but there are some risk factors associated with OSA development including growth, puberty, sex, and race [15, 112, 113]. Evidence that growth plays a part in the development of OSA comes from the age of onset frequently coinciding with rapid bone growth during puberty, tumour sites most frequently at the end of bones where active growth occurs, and people affected by OSA are on average taller than the unaffected population [15, 112]. There is a difference between sexes in the development of OSA with males more commonly affected than females, and there is some evidence of variation in the incidence rate between races in the USA [15, 112]. Although OSA itself does not appear to be directly heritable in most instances, there are heritable components to the risk factors [114].

There have been somatic mutations in tumour suppressor genes identified in individuals with heritable cancer syndromes, and there have been mutations identified in OSA tumours compared to non-tumours [109, 115–117]. There have, however, only been two somatic genetic mutations associated with OSA specifically [113]. This lack of identified somatic genetic associations is not surprising based on the lack of heritability observed in human OSA. Despite the lack of heritability and somatic genetic mutations identified there have been over 900 genes associated with human OSA [118]. These genes have been associated with OSA due to either differences in expression between tumour and non-tumour tissue, or due to mutations that have arisen in the tumour tissue compared to the non-tumour tissue [119–121]. Mutations in OSA tumour tissue, but not non-tumour tissue, and differences in gene expression between tumour and non-tumour tissue could be either the cause of the tumour or a result of the tumour. A possible cause of both differences in expression between tumour and non-tumour tissue, and mutations in tumour but not non-tumour tissue, is genomic and chromosomal instability [122, 123]. Genomic and chromosomal instability is a reported factor in many types of cancer progression [122, 123]. OSA has been shown to display chromosomal instability associated with mutations in the *TP53* gene [124]. A consequence of this chromosomal instability is aneuploidy, which can lead to the overexpression of some genes within malignant cells, disrupting normal cell processes [125]. Although mutations in *TP53* appear to be associated with chromosomal instability, the gene itself does not seem to be subsequently over expressed following aneuploidy [124, 125].

## Epidemiology and genetics of osteosarcoma in dogs

As outlined above, canine OSA is more common than human OSA [11, 12, 16]. Despite this there are similar risk factors associated with the development of OSA in dogs as in people, including growth and sex [16, 126]. In addition, OSA in dogs is also influenced by breed and neutering status [16, 126]. Large dogs constitute the majority of OSA cases [16, 94]. Similarly, people with OSA are commonly taller than average [112]. In the canine population, as with the human population, there also appears to be a sex disparity, with males more likely to develop OSA than females [12, 16]. Neutering status, although not relevant in the human context, appears to contribute to OSA risk where neutered dogs are more likely to develop OSA than non-neutered dogs [126]. This, combined with the association with puberty, suggests a complex role for sex hormone signalling in OSA risk. In contrast to human OSA, canine OSA appears to be heritable, with some breeds appearing to be predisposed to developing OSA compared to others [16, 127]. Interestingly of the 15 breeds with the highest reported incidence of OSA 12 are within a clear clade on the canine phylogeny [16, 128]. This relationship between affected breeds could indicate a potential common genetic origin of canine OSA across breeds, however the clade is large and contains many breeds that are not highly affected by OSA, thus this seems less likely [128].

Four breeds of dog that have a high incidence of OSA are the Rottweiler, Greyhound, Deerhound and Irish Wolfhound. The Irish Wolfhound, the largest breed, has the highest prevalence of OSA within the insured Swedish dog population once numbers within the population are taken into account [16]. It also has one of the lower median ages of onset at 6.6 years, only the greyhound has a lower median age of onset at 6.2 years [16]. Greyhounds are recognised to frequently develop OSA—accounting for around 25% of mortality in some cohorts [129, 130]. Although The Irish Wolfhound history includes a period when they were close to extinction [131]. In the process of conserving the breed the few remaining Irish Wolfhounds were crossed with Great Danes, Deerhounds, Borzois, and Mastiffs [131, 132]. This out-crossing will have introduced some genetic diversity, but to retain the Irish Wolfhound phenotype a large amount of inbreeding will have been required, therefore as with most modern breeds, genetic diversity is low within the breed [133]. Importantly for the Irish Wolfhound breed both Great Danes and Deerhounds have a high prevalence of OSA, and these were two breeds used to help rescue Irish Wolfhounds from extinction [16, 127, 131]. Combined with inbreeding, the use of two breeds predisposed to developing OSA could have led to Irish Wolfhounds acquiring OSA predisposition genes from both breeds. The Deerhound, a hunting dog, with references as far back as the third century; was split into various strains in the early nineteenth century, which is likely to have had an effect on genetic diversity. The Rottweiler is also a large breed of dog and within the insured Swedish dog population they have the 5th highest incidence ranking (after correction for actual numbers of dogs) for OSA with a median age of onset of 7.9 years [16]. The Rottweiler originated in Germany being used for guarding, droving, and as a draught dog [134]. Following the creation of breed standards in the 1930s, when the breed was registered with the UK and American Kennel Clubs it is likely that Rottweilers have exclusively been mated with other Rottweilers leading to a closed gene pool within the pedigree Rottweiler population [134, 135].

There have been 34 genetic loci associated with canine OSA across four breeds [136, 137]. One study identified 33 loci as associated with canine OSA across three breeds [136] and another identified a single locus associated with OSA in Deerhounds [137]. None of the loci were consistently associated across breeds, further suggesting that there may be a difference between breeds in the genetic predisposition to developing canine OSA [136, 137]. There have been 4 loci associated with the development of OSA in Irish Wolfhounds [136]. In Rottweilers 15 loci were associated with the development of OSA [136]. Deerhounds also have a high prevalence of OSA with an incidence rate of 15% and a heritability value of 0.69 [126, 127]. Those Deerhounds carrying putative high-risk dominant allele are considered to have a > 75% risk of developing OSA compared with those not carrying the allele. This has been mapped to CFA34 [137]. Unusually, in this breed, females appear to have an increased risk of developing OSA; this is not influenced by the neuter status [127]. Genetic analysis of Greyhounds has shown 14 inherited risk loci, the strongest in this breed was located 150 kb upstream of the tumour suppressor genes CDKN2A/B [136]. Interestingly the risk of developing OSA seems not to be a general feature of derivative breeds such as Whippets. Despite close clustering of molecular microsatellite markers, Whippets rarely suffer from OSA [138]. This raises the question of whether genetic factors alone account for the incidence of OSA in this breed. The influence of IGF-1 on size variation in dogs may have an influence in this regard, given the possible association with the development of canine OSA [139].

Currently none of the genetic variants identified as associated with canine OSA have been mechanistically verified. Verification of the mode of action could lead to the identification of novel therapeutic targets, and if these loci can be shown to consistently predict an increased risk of OSA, the number of affected individuals could be reduced. Breeders could genotype prospective sires and dams prior to mating and choose parents to reduce the risk of offspring having multiple deleterious OSA alleles in conjunction with minimising the epidemiological factors. In addition to the 34 genetic loci identified as associated with canine OSA, there have been genes identified as differentially expressed in canine OSA compared to non-tumour tissue that have implications for growth and metastasis, and are potential drug targets [140–143]. These genes have been identified utilising canine OSA tumour tissue, and canine OSA cell lines. There has also been variation in the expression of genes within tumours associated with survival time in canine OSA [97, 144–146].

## Conclusions

There are several dog breeds that appear to have a genetic predisposition to OSA for which no OSA associated loci have been identified, these breeds could benefit from the identification of OSA predisposition loci. Additional work is required to confirm the effect of the genetic loci identified as associated with canine OSA, and account for the variation observed in the development of disease. This is also the case with humans, therefore comparing the two species may provide valuable insights into disease origins and progression, given the many similarities between the tumour in both species. Further work is also required to establish improved treatment regimens for individuals that develop disease, the identification of particular genetic pathways that are altered in OSA tumour tissue compared to non-tumour tissue could facilitate this. Any genetic loci identified as associated with canine OSA have the potential to be examined for an association with human OSA, and any treatments shown to be effective in either species could be applied to the other.

## Abbreviations

HER-2: human epidermal growth factor 2 oncogene
OSA: osteosarcoma
SEER: surveillance, epidemiology, evidence and end results
VEGFA: vascular endothelial growth factor A
WHO: World Health Organisation
